# Issues of vaccination in premature infants: an overview

**DOI:** 10.1186/1824-7288-41-S1-A20

**Published:** 2015-09-24

**Authors:** Paolo Manzoni, Roberta Calzedda, Elena Altieri, Miguel Angel Pantoja Herrera, Maria Fioretti, Daniele Farina

**Affiliations:** 1 SC Neonatologia e TINO, Ospedale Sant'Anna, AOU Città della Salute e della Scienza, Torino, Italy; 2 Hospital Clínico San Borja Arriarán, Santiago, Chile

## 

Premature infants (PI) are neonates born <37 weeks gestational age, with different PI subgroups being identified according to gestational age and birth weight.

Prematurity is associated with increased morbidity and perinatal mortality, since yields increased risk for a number of pathological features and negative outcomes related mainly to the extent of all organs and functions' immaturity. Among these conditions, neonatal and post-natal infections play a major role. PI feature increased odds of infections and infections-related morbidity throughout their first months of life.

Among all preventative, anti-infective strategies, active vaccination is a key point for PI.

The most common vaccine-preventable diseases in PI are whooping cough, Haemophilus influenzae type-b (Hib) meningitis, invasive pneumococcal disease, rotavirus gastroenteritis, influenza.

International guidelines recommend to deliver active immunization following the chronological timing (=counting the weeks of actual birth), and not following the corrected gestational age (=counting the weeks of life starting from the expected moment of birth). However, vaccinations in PI are often performed later than recommended, or even less than expected, as shown by recent studies carried out in Italy assessing significantly decreased rates of active immunization in PI.

This somewhat poor adherence to the international guidelines is related to concerns about weaker immune responses in PI, and possible adverse events.

Nonetheless, several studies have shown that vaccines administered to PIs have excellent safety profiles, fully comparable to term infants.

Transient, benign episodes of apnea, with or without associated bradycardia, have been occasionally described in PI occurring up to 48 hours post-immunization. Though no significant morbidity nor long-term sequelae has been associated with these events, it is advisable to monitor these episodes for 48 hours after the completion of vaccination.

Vaccines are immunogenic, generally safe and well tolerated in all infants including PI. Noteworthy, only vaccines specifically authorized for use in premature infants should be used in PIs. The most immature neonates (i.e., ELBW infants) should receive their first dose of vaccine during hospitalization, in order to allow such risky groups of infants achieving sufficiently protective immunization before their discharge. This strategy also allows for adequate monitoring of cardiorespiratory function, and ultimately improves adhesion to the vaccination programs.

Strategies aiming at promoting education and awareness about vaccination practices and recommendations in PI should be reinforced. The ultimate aim is to increase delivery of effective protection against vaccine-preventable diseases to these vulnerable patients since their discharge from the NICU.

## Conflict of interest disclosure

All listed authors have no conflict of interest related to this article.
